# Supplementary material for: All-at-once RNA folding with 3D motif prediction framed by evolutionary information
Source: Nat Methods. 2025 Oct 3;22(10):2094–106. doi: 10.1038/s41592-025-02833-w (PMC12510878; doi:10.1038/s41592-025-02833-w)
Supplement: Supplementary file 3 — Source code to run CaCoFold-R3D. Data and results. [file 41592_2025_2833_MOESM3_ESM.gz › supplemental_material/results/CaCoFold-R3D/CaCoFold-R3D_Rfam/RF01987_ZEB2_AS1_4.R2R.sto.pdf]

RF01987_ZEB2_AS1_4

5′-G A Y A U U A A A C G C A U G G G G U C U A A G U U U U G R A G Y R U U U U U U U U U G A U A U U G C A A U U A A A R U A A A A U U A A A A A G A A A A
